# Lifestyle and environmental risk factors for unexplained male infertility: study protocol for Australian Male Infertility Exposure (AMIE), a case–control study

**DOI:** 10.1186/s12978-023-01578-z

**Published:** 2023-02-13

**Authors:** Sarah N. Biggs, Joanne Kennedy, Sharon L. Lewis, Stephen Hearps, Moira K. O’Bryan, Robert McLachlan, Simon von Saldern, Georgina Chambers, Jane Halliday

**Affiliations:** 1 Reproductive Epidemiology, Murdoch Children’s Research Institute, Melbourne, 3052 Australia; 2 Department of Paediatrics, University of Melbourne, Melbourne, 3052 Australia; 3 Department of Critical Care, University of Melbourne, Melbourne, 3052 Australia; 4 School of BioSciences and Bio21 Institute, Faculty of Science, University of Melbourne, Melbourne, 3010 Australia; 5 Clinical Andrology, Hudson Institute of Medical Research, Monash University, Clayton, 3168 Australia; 6 Healthy Male, Melbourne, 3004 Australia; 7 National Perinatal Epidemiology and Statistics Unit, University of New South Wales, Sydney, 2052 Australia

**Keywords:** ART, Male, Infertility, Idiopathic, Epidemiology, Lifestyle, Environmental exposure

## Abstract

**Background:**

Approximately 1 in 20 men are sub-fertile or infertile yet the aetiologies of male infertility remain largely unexplained. It is suggested that lifestyle choices and environmental factors contribute but research is limited. In particular, no study has evaluated early life exposures and subsequent male infertility. To address this knowledge gap, this study aims to characterise a cohort of men with idiopathic infertility and compare their general health, lifestyle choices and environmental exposures from teenage years onwards to men without reproductive abnormalities.

**Methods:**

Two groups of men (N = 500 cases; N = 500 controls), matched for age and socio-economic status, will be recruited from fertility clinics around Australia between June 2021 and June 2024. Men will be eligible if they are between 18 and 50 years, with a female partner less than 42 years, and have identified idiopathic male infertility (case) or are part of a couple with diagnosed female factor infertility but with no indication of compromised male fertility (control). Participants will complete an in-depth survey on general health, lifestyle and environmental exposures, reporting from teenage years onwards. An online medical data capture form will be used to gather fertility assessment information from participant medical records. Biological specimens of saliva (all study participants), blood and urine (optional) will be collected and stored for future genetic and epigenetic analysis. Differences in outcome measures between cases and controls will be determined using appropriate between groups comparisons. The relationship between explanatory variables and infertility will be analysed using multilevel modelling to account for clustering within fertility clinics.

**Discussion:**

This study addresses an important gap in research on the aetiology of male infertility and will provide a comprehensive profile of the lifestyle and environmental risk factors for male infertility, leading to provision of up-to-date health advice for male teenagers and adults about optimising their fertility.

## Plain language summary

Approximately 1 in 20 men are sub-fertile or infertile yet very little is known about the causes of male infertility. Research has suggested that lifestyle choices and environmental factors contribute to infertility, but more needs to be done to identify and verify the full suite of associations.

We will recruit up to 1000 Australian male partners within couples who are seeking help from fertility clinics to get pregnant. They will be asked about their general health, lifestyle and environmental exposures at home or work over their lifespan. We will compare findings between men who are sub- or infertile with men who are not. Any differences will help us understand what factors may be associated with risk of infertility in men.

This study will provide important information to clinicians and to inform public policy that will lead to prevention and improved treatment strategies for infertile men. The data gathered from this study will enable future research including the genetic and epigenetic basis of male infertility.

## Background

Infertility affects up to 15% of couples of reproductive age [1] with male factors contributing to 30–50% of infertility cases, representing approximately 1 in 20 men in western societies [2–4]. In Australia and New Zealand, known causes, such as genetic changes, injury or illness, account for under a third of sub- or infertile men however the precise cause in 77% of men in couples seeking ART is unknown [5].

While poorly quantitated, at a social and financial level, male infertility is a highly significant medical problem. It contributes to the growing use of assisted reproduction technology (ART) and substantial costs to individuals and the health system [6]. In addition, there are data to indicate that infertile men have a significantly higher incidence of comorbidities than fertile men. These include metabolic disorders, cancers of several types, inflammatory disease and of overall premature mortality [7–10]. As such, a better understanding of the aetiology of male infertility will inform male health promotion strategies and general health management.

Increasingly there is recognition that some male infertility is the result of lifestyle or environmental factors [11–13]. Diets high in processed meats, red meat, soy, simple carbohydrates and full-fat dairy have been negatively associated with both sperm quality and pregnancy outcomes [12]. It is known that heavy smoking, adiposity, use of androgenic steroids, alcohol dependence and drug use can impair sperm count, motility, morphology and sexual function [13]. To a lesser degree, endocrine disrupting chemicals such as bisphenol A (BPA) and phthalates, pesticides/herbicides, heavy metals, air pollution and electric magnetic frequencies via mobile phones and computers have also been associated with reduced sperm concentration, volume, motility and viability [14]. The results of these exposures are not consistent across studies, which are often cross-sectional, collecting data for a single timepoint, usually at the time of ART use. There is limited evidence regarding whether these lifestyle and/or environmental exposures impair male fertility when the exposure happens earlier in life, when consideration of a future family may not be at the forefront [11].

The current limited knowledge on the link between lifestyle and environmental exposures over the lifespan has resulted in a paucity of health promotion, preventative strategies and treatment options for male infertility [15, 16]. This study will address this gap in knowledge by recruiting case and control groups and collecting lifespan data on exposures related to physical health, lifestyle and the environment, through a detailed survey and provision of biological specimens.

### Methods/design

**Fig. 1:**
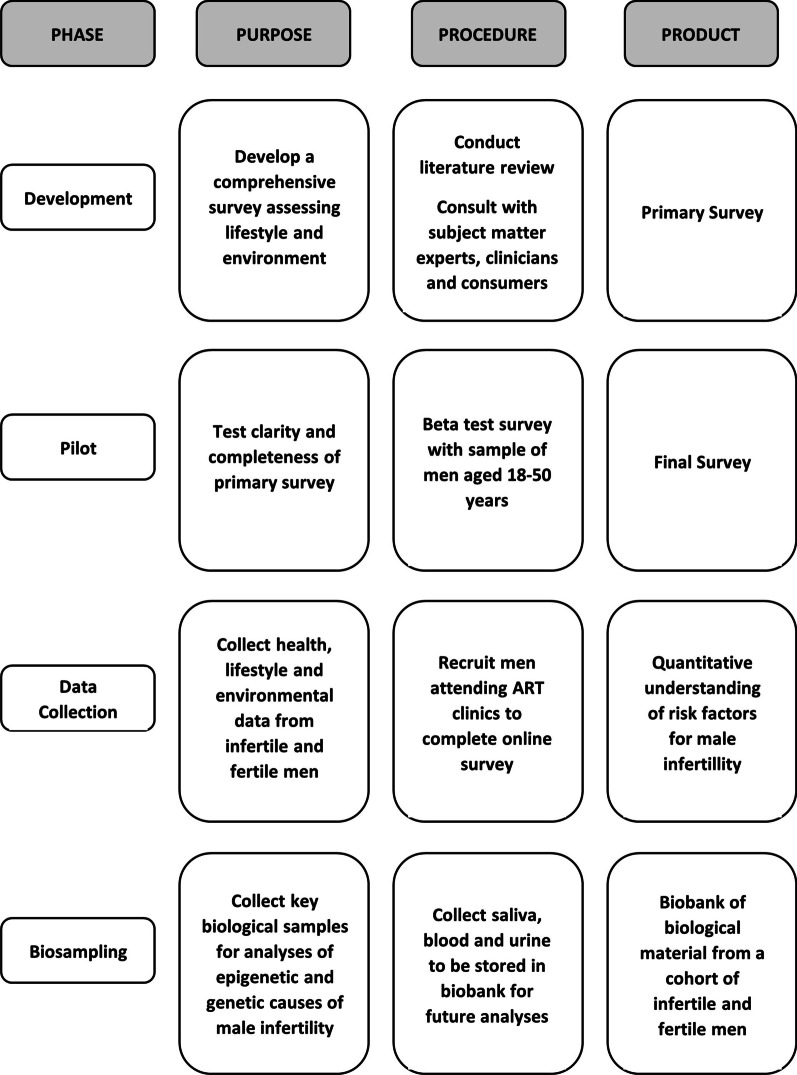
Diagrammatic summary of AMIE research protocol

A summary of the research procedure is presented in Fig. 1. Full details are outlined below.

### Aims

The specific aims of this study are to:

1. establish a population of men with idiopathic male infertility (cases) and with normal fertility parameters (controls) across multiple Australian fertility clinics;
2. describe the health burden of infertile men and compare with that of fertile men via the self-report survey;
3. identify risk factors for male infertility from a survey completed by cases and controls on lifestyle and environmental exposures since their teenage years;
4. collect key biological samples for future molecular analysis to investigate genetic and epigenetic causes of male infertility and possible interactions with lifestyle and environmental exposures.

### Study design

Case control study.

### Study setting

Australian fertility clinics accredited to perform assisted reproductive technology (ART) were selected as recruitment sites for cases and controls based on national representation and reported number of eligible patients accessing their services annually. Sites from Queensland, New South Wales, Victoria, and Western Australia with eligible patient throughput greater than 300 per annum were approached to participate.

Data collection, management, and analysis will be performed by the Murdoch Children’s Research Institute (MCRI), located at The Royal Children’s Hospital in Melbourne, Australia.

### Study timeline

The overall duration of the study is four and a half years, with the final year being allocated to analysis and dissemination of results. Recruitment of fertility clinics and submission of ethics applications began in 2020. Recruitment and data collection for the study is being undertaken in 2021–2024 with data analysis to commence mid-2024.

### Ethics approval

Ethics and/or governance approval was granted by The Royal Children’s Hospital Human Research Ethics Committee (HREC/69249/RCHM-2020). Site specific governance was obtained from seven fertility clinics in Victoria, New South Wales, Queensland and Western Australia. Further sites can be added as required.

### Eligibility criteria

Eligible men must be attending one of the collaborating fertility clinics for fertility assessment and potential provision of ART, be between 18 and 50 years with a female partner less than 42 years and capable of providing informed consent in English.

### Inclusion criteria

Participants must meet the following criteria to be enrolled in this study:

- Case: has identified infertility of unknown origin, either as male factor only (i.e. the female partner is not infertile) or as mixed factor where the male infertility is of unknown origin and his female partner has known cause of infertility (tubal disease, ovulatory disorder or endometriosis).
- Control: is attending the ART service as part of a couple with diagnosed female factor infertility of known cause but with no identified male infertility and normal semen analysis (fertility assessment normal).

### Exclusion criteria

Participants meeting any of the following criteria will be excluded from this study:

- Male Infertility of a known aetiology such as genetic disorders, cancer treatment, testes trauma, or obstructive azoospermia

### Recruitment procedures

The identification of eligible men from daily site attendance lists will be performed by the fertility clinic representative as per specific site protocols. A minimum dataset on each eligible man will be recorded via a secure online database developed in REDCap [17, 18]. These details will include: ART site, year of birth, postcode and categorisation as case or control. This is designed to capture minimum information on all eligible cases and controls to determine sample representation.

**Fig. 2:**
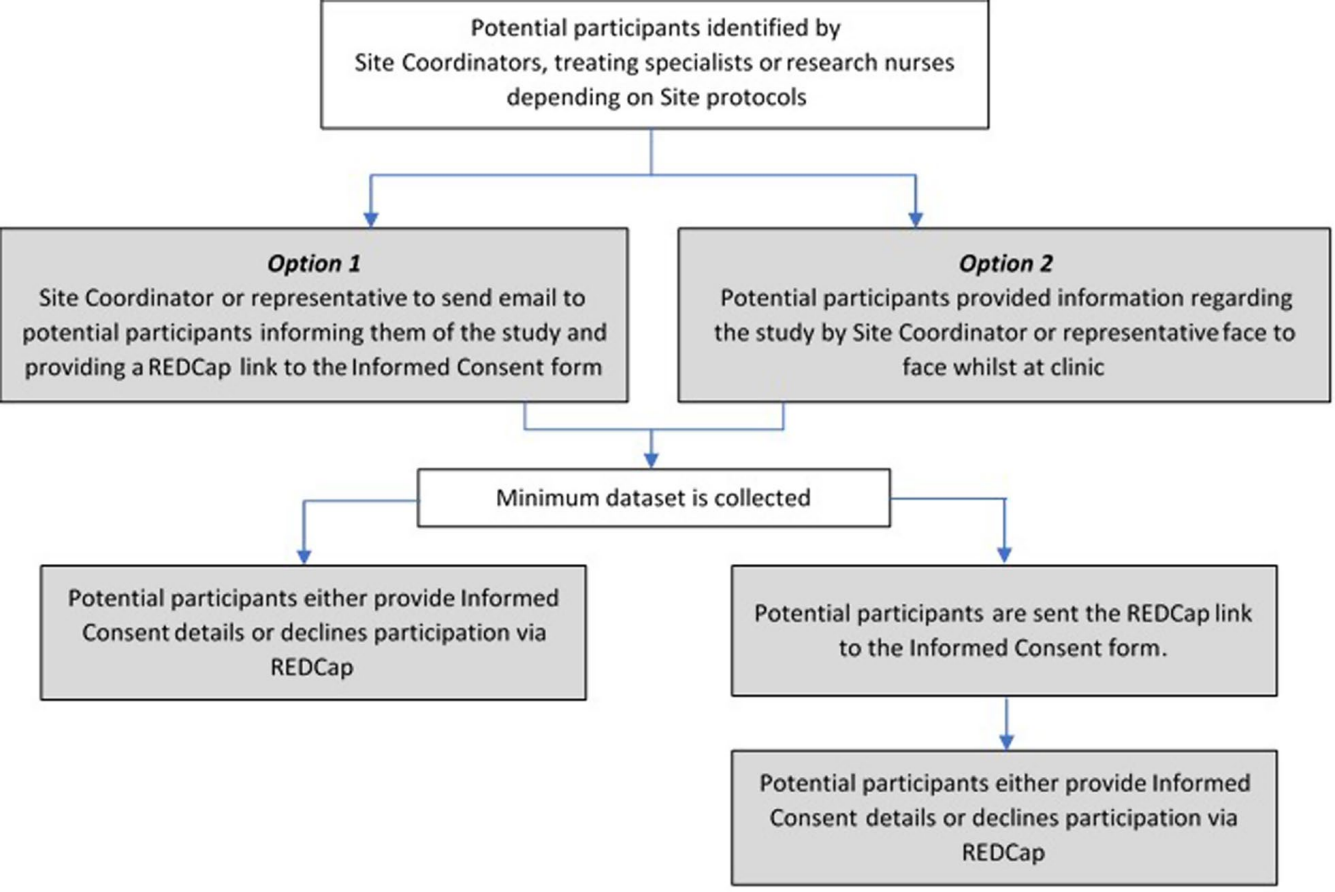
Recruitment flowchart with options dependent on ART site requirements

The clinician or site representative will approach the men at one of three time points during their standard care appointments and provide information regarding the study and participation (see Fig. 2):

- At reception, upon check in for their clinical appointment;
- In the doctor’s rooms when treatment plans are prepared; or
- Via email prior to their clinical appointment or commencement of treatment.

Patients will be asked to view the study information and provide informed consent through the REDCap database. If they decline participation, they will be asked if they are willing to disclose why, and this will be entered into the secure database.

If patients agree to participate in the study, they will be presented with an informed consent document that includes agreement to:

- Complete the survey
- Allow fertility assessment results to be disclosed to the AMIE study team from fertility clinics
- Provide a saliva sample
- Allow their data to be extracted from the Australian and New Zealand Assisted Reproductive Database (ANZARD) so that treatment information and pregnancy outcomes can be provided to the AMIE research team in the future.

ANZARD is a Clinical Quality Registry comprising information on all ART treatment cycles undertaking in Australia and New Zealand fertility clinics. ANZARD is managed by the National Perinatal Epidemiology and Statistics Unit of the University of New South Wales, Sydney [19]. Fertility Clinics are required to submit data to ANZARD as part of their licencing requirements and thus full ascertainment of detailed information on all ART treatment cycles and their outcomes is achieved. ART treatment data will be available for men recruited into the AMIE study from January 2024.

A further optional consent will be sought for collection of blood and urine to be stored for future genetic and epigenetic analyses.

## Measures

### AMIE survey

#### Development

A working party was convened to develop the in-depth survey to be used in this study. The working party consisted of experts in ART research and clinical practice, epidemiologists, and representatives from a professional body (Victorian Assisted Reproductive Treatment Authority) and consumers (Healthy Male, Australia). The survey was piloted for clarity and completeness with a convenience sample of 25 men aged 18–50 years. It is unknown whether any of the pilot sample had sub- or infertility.

Table 1 shows the sections, outcomes or exposures and variables of interest that will be collected through the AMIE survey.

**Table 1:** The sections, outcomes and self-reported data gathered from participants through the AMIE survey

Section name	Outcome / exposure	Variables
About You	Demographic information	Age, education, employment status, ethnicity, length of time in Australia if born overseas
General Health (1) (Objective 2)	Perinatal and childhood medical history	Perinatal outcomes, maternal smoking during pregnancy, physical disability, childhood illness
General Health (2) (Objective 2)	History of health and chronic disease from teenage years	Chronic physical and mental health conditions or illness, medication use, fertility related conditions (e.g. varicocele), history of surgery, history of hospitalisations, sexually transmitted infections, weight and body shape
Fertility history (Objective 2)	Family history of infertility Personal history of infertility	Method of conception and pregnancy outcomes of offspring from male blood relatives Method of conception and pregnancy outcomes of any previous offspring from AMIE participant
Lifestyle (Objective 3)	History of lifestyle factors from teenage years	Diet, exercise, use of hot baths, spas and saunas, choice of underwear, alcohol use, smoking, medication and drug use, sleep and stress
Environmental exposure (Objective 3)	History of exposure to environmental toxins from teenage years	Exposure to phthalates, BPAs, parabens, insecticides, toxic chemicals, heavy metals, radiation, temperature of physical pressure and electromagnetic fields

To address the paucity of information regarding exposures from childhood to adulthood [11], questions were designed to capture retrospective data on lifestyle and environmental exposures from teenage years onwards. Preliminary analysis of the first 25 completed surveys shows an average completion time of 38.3 ± 17.1 min (median = 35: range 17–91 min).

### Medical data

An online medical data capture form, hosted on the secure REDCap database [17, 18], will be used to store manually extracted available fertility assessment data from participant medical records conducted as part of the ART site’s routine clinical care. The medical data capture form for fertility assessments has four sections. However due to the difference in clinical assessments across ART sites, it will not be compulsory to complete all sections. The ART clinics have been asked to provide any information available that fall into these four areas:

1. Physical exam (e.g. testicular volume, presence of gynaecomastia, varicocele, hydrocele, vas deferens)
2. Hormone analysis (e.g. FSH, LH, testosterone)
3. Semen analysis (e.g. volume, sperm concentration, motility, morphology)
4. Genetic testing (e.g. karyotyping, Y-chromosome microdeletion)

Fertility assessment and infertility treatment data will be obtained from the ART clinics 12 months following the participants enrolment into the study and ANZARD thereafter.

### Biosampling/biomarkers

We will ask participants to provide saliva, and as an option, peripheral blood and urine samples to be stored for future analysis (objective 4). Additional consent will be sought for the use of data and samples in future research projects, approved by the HREC, that are an extension of, or closely related to, the original project. Participants themselves will collect the saliva sample, while the peripheral blood and urine samples will be collected at the recruiting sites or at a public pathology provider located locally to the participant.

Examples of future analysis of the biosamples collected include:

Saliva and blood:

- Epigenetic analysis including DNA methylation
- Genetic analysis to determine genetic causes of male infertility

Blood:

- The presence and concentration of phthalates, lead, mercury, cadmium and oxidative stress
- Metabolomic profile

Urine:

The presence and concentration of parabens, persistent organic pollutants, other endocrine disrupting chemicals (e.g. solvents, propellants, aerosols), insecticides/herbicides/pesticides, markers of oxidative stress and bisphenols.

### Addressing potential bias

#### Recruitment and participation bias

The minimum data will be collected from all eligible patients to compare the proportion and characteristics of responders and non-responders, and cases and controls among fertility clinics.

#### Reporter bias

The survey contains a question regarding the participants perception of the causes of male infertility. Participants are asked to list what they think are the top five causes of male infertility from 16 choices which include health, lifestyle and environmental factors. They are also able to write free text responses for anything else they consider important. These responses will be regressed against targeted variables to determine if reported perceptions predict responses on exposure variables.

#### Location bias

The number of patients who meet the eligibility criteria and demographics of each fertility clinic will vary. Therefore, modelling will include site as a clustering variable to address location bias.

### Sample size

According to figures reported by the participating fertility clinics, of all the men in couples seeking ART (N =  ~ 3700/y), 20% will have unexplained male factor infertility (N =  ~ 740/y) and 50% will be medically fertile (N =  ~ 1850/y). Based on recruitment rates in previous studies [20, 21], it is anticipated that 30–40% of identified cases (N =  ~ 260/y) and 15% of identified controls (N =  ~ 278/y) will provide informed consent. We also estimate a 10% loss to follow-up through not completing the questionnaire. We will endeavour to approach all eligible men over the recruitment period to ensure we meet our sample targets.

With a ratio of 1:1 cases and controls and a baseline prevalence rate of 2% for an environmental exposure of interest, such as working with motor mechanics [22, 23] a sample of 500 in each group would find an increase in prevalence to 5.5% as significant with 80% power and p < 0.05. Similarly, a baseline prevalence of lifestyle exposures in the general population, such as non-injecting drug use (3–4%) [24], if increased to 10% in cases, would be significant with N = 285 in each group. Early data analysis is suggesting this degree of difference is present. A sample size of 500 in each group is feasible based on our estimates and a recruitment period of 3 years.

### Statistical analysis

All analyses will be conducted using the latest version of Stata [25]. All continuous data will be examined for normality and transformed using standard transformation techniques if required. The representativeness of the participating sample will be tested using the minimum dataset of the study population. Other biases will be tested as mentioned above. The continuous demographics, age and socio-economic status determined by SEIFA scores, will be compared between cases and controls via independent samples *t*-tests. Differences in categorical demographic variables will be examined using Chi-squared tests.

Chi-squared or *t*-test analyses will be conducted to determine initial differences between cases and controls on health, lifestyle and environmental exposures. Environmental exposures will be categorised as ‘ever’ or ‘never’. Where the exposure is present in at least 15 cases and 5 or more controls, exposures will be categorised into low, moderate or high depending on inter-quartile ranges. The dose–response relationship between exposures and infertility will then be examined.

The relationship between explanatory measures and infertility will be analysed using multilevel modelling (MLM), to account for ART site clustering. These models will be extended to determine the risk of environmental exposures on infertility. Covariates will be included in the models if they are associated with the outcome at p ≤ 0.1 in the bivariate models. Testing for collinearity and log likelihood ratios will be used to determine the model(s) of best fit.

## Discussion

This case–control study will identify the health burden of and the lifestyle and environmental risk factors for male infertility. The results will provide biopsychosocial profiles to inform future male infertility prevention and management.

With consent in place, the study will also provide a valuable cohort of men with idiopathic infertility and matched controls about whom long-term and reproductive outcomes can be obtained through linkage with ANZARD.

There are several strengths of this study. These include: the large sample size and national representation, controls recruited from the same source population and using the same survey methodology for data collection. To minimise the impact on clinical resources and optimise participant engagement, we have developed bespoke procedures for each ART site which gives us the ability to integrate recruitment into the clinic’s workflow. The survey will provide a broad self-reported collection of lifestyle and environmental exposure information across the lifespan, thereby enabling the development of at-risk profiles. In addition, biological samples will be collected and available for analysis for DNA markers associated with male infertility and for objective measures of self-reported environmental exposures.

Recruitment to date has been challenging due to the ongoing lockdowns and restrictions put in place as a result of COVID-19. These have meant that patient appointments have been conducted virtually or delayed. As such, patients have not been exposed to study information via the usual methods while waiting in reception areas of fertility clinics. To adapt to these changing conditions, we produced a study information video, hosted on YouTube. The link to this video is emailed to patients with a request to participate. The video is also presented on a tablet device in waiting rooms, replacing, or as an adjunct to, the traditional paper brochures, when face-to-face appointments recommence.

Aside from the pandemic, other limitations include variable amount of fertility assessment data available across ART sites due to different clinical assessment protocols at each site; the sensitivity of the topic may be limiting agreement to participate, especially for infertile men who may be particularly concerned about privacy issues; potential recall bias, particularly with the older participants who are asked to report on longer historical periods than the younger participants.

Nonetheless, this study addresses an important gap in research on the aetiology of male infertility, focusing on data pertaining to teenage years and beyond about general health, lifestyle habits and environmental exposures in both infertile and fertile men. Accompanied with biospecimen data, this study will provide a comprehensive profile of the risk factors for male infertility, leading to provision of up-to-date health advice for male teenagers and adults about optimising their fertility. This information will be disseminated through their clinical carers, professional bodies, social networks and the media.

## Data Availability

No applicable.

## Abbreviations

AMIE: Australian Male Infertility Exposure
ANZARD: Australian and New Zealand Assisted Reproductive Database
ART: Assisted reproductive technology
BPA: Bisphenol A
HREC: Human Research Ethics Committee
MCRI: Murdoch Children’s Research Institute
MLM: Multi-level modelling
SEIFA: Socio-economic indexes for areas
